# Cereal and Cereal Products: Quality, Functionality, Health Security and Application of New Technologies

**DOI:** 10.3390/foods15081280

**Published:** 2026-04-08

**Authors:** Olivera Šimurina, Elizabet Janić Hajnal

**Affiliations:** Institute of Food Technology, University of Novi Sad, Bulevar cara Lazara 1, 21000 Novi Sad, Serbia; elizabet.janich@fins.uns.ac.rs

## 1. Introduction

Global food systems are currently facing unprecedented challenges driven by population growth, climate change, resource limitations, and evolving dietary patterns. Ensuring a stable supply of safe, nutritious, and sustainable foods has therefore become one of the central priorities of modern food science [1]. Within this context, cereals and cereal-based products play a fundamental role, as they represent one of the most important sources of dietary energy and nutrients for populations worldwide [2,3]. At the same time, advances in food technology, processing innovations, and sustainability-oriented production strategies are transforming the cereal sector and opening new opportunities for the development of healthier and more resilient food systems [2,3,4].

Cereals and cereal-based products occupy a central role in human nutrition, serving as staple foods that underpin global food security and public health [1,2]. For centuries, cereals have formed the basis of traditional diets and remain among the most widely consumed food commodities worldwide [1]. Traditionally recognized as primary sources of carbohydrates, cereals are now increasingly appreciated for their broader nutritional contributions, including dietary fiber, proteins, essential vitamins, minerals, and numerous bioactive compounds [2,5,6]. As dietary habits evolve and consumer awareness of nutrition continues to grow, research efforts have increasingly focused on improving the quality, functionality, and safety of cereal-based foods [5,7]. In recent years, the development of cereal products with enhanced nutritional profiles and functional properties has emerged as a major research priority in food science and technology [2,6,7]. These developments reflect the growing demand for foods that support both human health and sustainable food systems [2].

Recent advances in analytical methods and food processing technologies have significantly improved the characterization and utilization of cereal grains [2,4,5]. Techniques such as rheological analysis, advanced spectroscopic methods, and high-resolution imaging enable detailed evaluation of the structural and physicochemical properties of cereals [4,5,8]. Such analytical approaches provide deeper insight into the complex interactions between cereal components and technological processes, which ultimately determine the quality of cereal-based foods [4,5].

At the same time, innovative processing approaches—including fermentation, enzymatic bioprocessing, and non-thermal technologies—are increasingly applied to improve nutrient bioavailability, sensory quality, and product safety [7,9,10,11,12]. Further, recent reports position 3D printing, nanotechnology, and selective novel processing methods as key enablers for tailoring cereal product quality, functionality, and safety [13,14,15,16,17]. These technological developments open new opportunities for designing cereal-based foods that combine improved nutritional value with desirable technological and sensory characteristics [4,6,7,9,15,16,18].

Despite these advances, the cereal food system continues to face several challenges. The presence of mycotoxins and other contaminants remains a major concern for food safety, while climate variability further complicates risk management strategies [8,19]. Environmental changes, particularly fluctuations in temperature and humidity, may significantly influence fungal growth and the occurrence of mycotoxins in cereal grains [20].

In addition, emerging environmental contaminants such as per- and polyfluoroalkyl substances (PFASs) and trifluoroacetic acid (TFA) have raised new concerns regarding food safety and environmental exposure [21]. These developments highlight the need for integrated monitoring strategies and more robust regulatory frameworks to ensure the safety of cereal-based foods [20,21].

Beyond current technological approaches, future progress in cereal science will increasingly depend on the integration of advanced digital and sustainability-oriented technologies [22,23]. Artificial intelligence (AI) is expected to play an important role in cereal research and agri-food systems by enabling predictive modeling of grain quality, optimization of processing conditions, and improved quality management throughout the food supply chain [19,22,23,24,25].

The Special Issue “Cereal and Cereal Products: Quality, Functionality, Health Security and Application of New Technologies” was therefore initiated to address key challenges and knowledge gaps in cereal science, particularly those related to improving cereal product quality, enhancing nutritional functionality, strengthening food safety, and promoting the application of innovative technologies.

## 2. An Overview of Published Articles

Nine manuscripts were submitted for consideration for the Special Issue, and all of them were subject to the rigorous *Foods* review process. In total, five articles were finally accepted for publication and inclusion in this Special Issue.

The article by Nićetin et al. [26] investigates the formulation and quality optimization of cookies enriched with celery root powder. Using a systematic approach, the authors assessed the impact of varying concentrations of celery root powder on the physical, chemical, and sensory properties of the cookie products. The results show that adding celery root powder improved dietary fiber content and antioxidant activity while maintaining acceptable sensory attributes. The optimal formulation balanced health benefits and consumer acceptability, demonstrating the potential of vegetable-based ingredients in functional bakery products. Their research demonstrates that integrating unconventional vegetable-based ingredients can significantly improve the nutritional profile (e.g., increased fiber and antioxidants) of cereal products without compromising sensory acceptance. This investigation addresses the gap in developing functional, health-promoting baked goods using novel raw materials.

The second article by Šimurina et al. [27] explores the phytochemical stability and antioxidant activity in “proja,” a traditional Balkan baked dish, when prepared using pigmented maize varieties. The research analyzes the retention of bioactive compounds, such as phenolics and anthocyanins, after baking. Findings indicate that pigmented maize retains significantly higher levels of phytochemicals and antioxidative capacity in the final product compared to conventional maize. The study highlights the nutritional enhancement of traditional foods through the use of pigmented cereals, promoting both cultural heritage and health benefits. Their findings reveal that pigmented maize varieties retain higher levels of beneficial compounds post-baking, promoting the use of traditional foods with enhanced health benefits. This work advances understanding of how ingredient selection and processing affect the final nutritional quality of cereal-based dishes.

Research by Maoloni et al. [28] presents the development of a novel non-alcoholic beverage based on einkorn wheat, produced through lactic acid fermentation. The study evaluates the microbiological safety, chemical composition, and sensory properties of the beverage. Results demonstrate that lactic fermentation enhances the safety and nutritional profile, particularly increasing bioactive compounds and desirable organic acids. Sensory analysis confirms good consumer acceptance. The work supports the innovation of cereal-based functional beverages leveraging ancient grains and fermentation. Their comprehensive assessment of microbiological safety, chemical characteristics, and sensory properties illustrates how fermentation technology can transform ancient grains into innovative, health-oriented beverages. This fills a gap in research on expanding the use of traditional cereals through modern processing methods.

The article by Lou et al. [29] examines the domestication and cultivation of the edible mushroom *Pholiota adiposa*, particularly focusing on strains that adapt to high temperatures. The study emphasizes the effective use of agricultural residues as substrates, thereby enhancing the circular economy and sustainability. Additionally, the nutritional analysis shows that co-cultivation with grains enriches the nutritional profile of both the mushroom and the associated grains. The findings underline the dual benefits of sustainable agricultural practices and improved food nutrition in regional settings.

Finally, Dvořáček et al. [30] investigate the effects of incorporating grape pomace—a by-product of the wine industry—on the phenolic composition and dough properties of wheat buns. The results show that grape pomace enrichment significantly increases the polyphenol content and antioxidant activity of the final product, supporting the valorization of agro-industrial by-products in functional cereal-based foods.

## 3. Conclusions

The articles included in this Special Issue provide valuable insights into recent developments in cereal science and technology. Collectively, they demonstrate how advances in processing technologies, ingredient innovation, and sustainability-oriented approaches contribute to the development of improved cereal-based foods.

Although substantial progress has been made in cereal quality assessment, processing technologies, and contaminant monitoring, important knowledge gaps remain. More research is needed to better understand the long-term health implications of cereal products produced using emerging processing technologies.

Sustainability considerations, especially comprehensive life cycle assessments comparing conventional and innovative cereal processing methods, also remain insufficiently explored. Furthermore, limited knowledge regarding the environmental pathways, bioaccumulation, and dietary exposure to emerging contaminants represents an additional challenge for the cereal sector.

Future advances in cereal science will increasingly depend on the integration of innovative technologies, improved analytical tools, and interdisciplinary research approaches. Continued progress in these areas will be essential for the development of safe, nutritious, and sustainable cereal-based foods capable of supporting resilient global food systems and improving public health worldwide.

## Data Availability

Not applicable.
